# Publication reform to safeguard wildlife from researcher harm

**DOI:** 10.1371/journal.pbio.3000193

**Published:** 2019-04-11

**Authors:** Kate A. Field, Paul C. Paquet, Kyle Artelle, Gilbert Proulx, Ryan K. Brook, Chris T. Darimont

**Affiliations:** 1 Department of Geography, University of Victoria, Victoria, British Columbia, Canada; 2 Raincoast Conservation Foundation, Sidney, British Columbia, Canada; 3 Alpha Wildlife Research and Management, Sherwood Park, Alberta, Canada; 4 Department of Animal and Poultry Science and the Indigenous Land Management Institute, University of Saskatchewan, Saskatoon, Saskatchewan, Canada

## Abstract

Despite abundant focus on responsible care of laboratory animals, we argue that inattention to the maltreatment of wildlife constitutes an ethical blind spot in contemporary animal research. We begin by reviewing significant shortcomings in legal and institutional oversight, arguing for the relatively rapid and transformational potential of editorial oversight at journals in preventing harm to vertebrates studied in the field and outside the direct supervision of institutions. Straightforward changes to animal care policies in journals, which our analysis of 206 journals suggests are either absent (34%), weak, incoherent, or neglected by researchers, could provide a practical, effective, and rapidly imposed safeguard against unnecessary suffering. The Animals in Research: Reporting On Wildlife (ARROW) guidelines we propose here, coupled with strong enforcement, could result in significant changes to how animals involved in wildlife research are treated. The research process would also benefit. Sound science requires animal subjects to be physically, physiologically, and behaviorally unharmed. Accordingly, publication of methods that contravenes animal welfare principles risks perpetuating inhumane approaches and bad science.

## Abstract 2

An analysis of the “instructions for authors” for wildlife journals reveals deficits in animal care policies but identifies a transformative path towards ethically attuned treatment of wild research animals.

Scholarly journals can shape the behavior of researchers towards improved science and adherence to ethical standards. Editorial policies demanding open data and improved transparency, for example, encourage increased reproducibility in science [1–3]. Likewise, improving editorial standards for the just treatment of laboratory animals has come into sharp focus [4, 5]. The welfare of vertebrates studied in the wild, however, has received less consideration despite the reality that wildlife research primarily occurs outside the direct supervision of research institutions. Moreover, whereas laboratory animals belong to only a handful of taxa, wildlife research can span physiological and behavioral variation across more than 60,000 vertebrate species [6].

Herein, we describe how inattention to the just treatment of wildlife poses a significant problem in research ethics but identify a potentially transformative route towards change. Analyzing data on animal care policies across 206 journals that commonly publish conservation and wildlife research, we reveal not only striking variation across journals and significant shortcomings of editorial oversight but also unrealized potential for change. Informed by the patterns we reveal, we advocate for more robust animal care policy and strong enforcement by editors, reviewers, and scholarly societies with the aim of rapid change. Given that publishing is at the core of the academic reward system, we believe that this approach offers a practical and easily applied protection against mistreatment of wild research animals. To provide broader context and to explain our focus on animal care policies, we also offer illustrative examples of how other layers of oversight, including legal and institutional, can fall short of safeguarding wildlife from researcher harm.

## Why “wildlife welfare” matters in wildlife science and conservation

Science provides information; it does not justify unethical behavior. Accordingly, values matter in the research process. In an agenda-setting article for the field of conservation biology, for example, a set of proposed postulates—a “normative set”—to systematize the field was centered on value-laden assumptions (e.g., “Biotic diversity has intrinsic value”) [7]. Similarly, animal welfare science incorporates value-based assumptions about the moral significance of animals and their quality of life [8]. More broadly, the ethical appropriateness of animal suffering and killing in research has encouraged compelling scholarly dialogue and debate [9–12]. Some have argued that employing wildlife research methods without due consideration for the well-being of wildlife (herein referred to as “wildlife welfare”) is ethically untenable (e.g., [11]). Although academic attention to wildlife welfare has been considerable, practical solutions towards rapid change (such as publication reform, for which we advocate here) are rare.

Failure to consider wildlife welfare [13] during research can harm not only individual animals but also the scientific process. In terms of harming animals, for example, Waugh and Manomy [14] highlight how lethal sampling of free-swimming minke whales (*Balaenoptera bonaerensis*) via harpooning led to slow rates of death, suffering that could have been prevented with available nonlethal research approaches. Even nonlethal approaches, however, can cause harm. Capture and tagging of wildlife, common activities in wildlife research, can impose physical handicaps that can last days to months, potentially compromising welfare, behavior, fitness, and the reliability of data [13, 15]. Beyond animal welfare, sound science requires that animal subjects be unencumbered physically, physiologically, and behaviorally by harm; an individual’s altered state can affect experimental and observational validity, reliability, and replicability [16]. Accordingly, research methods that contravene animal welfare principles risk not only inhumane treatment of animals but also low-quality science.

## Existing oversight mechanisms and their shortcomings

Legal levels of oversight in theory provide enforceable direction to researchers but can fail. In some countries, the application of legislation to the care and use of wild research animals is not particularly clear (e.g., [17]). For example, the United States Animal Welfare Act predominantly oversees the care and use of captive animals and excludes studies from Institutional Animal Care and Use Committee (herein referred to as animal care committee) review if the study “…does not involve an invasive procedure, and which does not harm or materially alter the behavior of the animals under study.” This exemption could compromise animal welfare; the terms “invasive,” “harm,” and “materially alter” are not defined and thus are exposed to variable and subjective interpretation by researchers depending on their expertise, experience, and knowledge of physiological and behavioral indicators of pain and suffering [6, 17]. Such uncertainty might be especially relevant to wildlife, which typically cannot be monitored as closely for harm as laboratory animals. Moreover, activities that cause mortality and morbidity, such as chemical immobilization [18], banding [19], and capture [13], have been classified by some government agencies as not invasive, harmful, or materially altering of behavior, thereby granting them exemption [17]. Beyond the ambiguity of what constitutes harmful methods in the US Animal Welfare Act, no legislation in the US governs who should perform surgeries on wild research animals, which have potential to be damaging if not executed meticulously [17]. By contrast, the European Union’s Directive 86/609/EEC on the protection of animals used for scientific purposes regulates the use of animals, including (and explicitly) wildlife, through a systematic project evaluation requiring assessment of pain, suffering, distress, and lasting harm imposed on research animals [20].

Legal oversight can also vary within countries, which might limit clarity if fieldwork is conducted remotely from academic institutions. In Canada, the federal government does not have the jurisdiction to legislate research involving animals. Instead, animal care in research falls under provincial jurisdiction, which lacks regulatory consistency across the country. For example, whereas some provinces have legislation overseeing animal care in research (e.g., Nova Scotia), others merely endorse regulations (e.g., Alberta) [21]. Moreover, national standards for animal care in research, such as those suggested by the Canadian Council for Animal Care (CCAC), are inconsistently incorporated into provincial jurisdictions across the country (only five provinces refer directly to the CCAC) [21]. By contrast, the *Australian code for the care and use of animals for scientific purposes 8th edition 2013*, which applies to all live nonhuman vertebrates and cephalopods, has been incorporated under each state and territory’s animal welfare legislation, making compliance mandatory in each jurisdiction.

Recognizing relevant legal frameworks and other sources of guidance, institutions serve a more direct role in oversight of their researchers. As do other scientists, wildlife researchers seek approval from an animal care committee for their research because it is required by their university, often the funding agencies supporting their research, and/or target journals in which they plan to publish their results. Owing to the profound differences between wildlife and laboratory animals [6], however, animal care committee members might lack wildlife-related expertise, rendering assessments inadequate to ensure wildlife welfare [17]. The *Guide for the Care and Use of Laboratory Animals* [22] (the Guide) is a standard mandated by the 1986 Public Health Service Policy in the US to aid animal care committees in their reviews. Although recent revisions to the Guide incorporated consultation with wildlife biologists, a recent assessment considered the consultation to have occurred in a “cursory and broad manner” [6]. Taxon-specific organizations are engaging in outreach to animal care committees to address this potential inadequacy. For example, the American Society of Mammalogists developed a protocol form for Institutional Officials and animal care committee chairs designed specifically for wildlife research conducted either in the field or in captivity [6]. Despite these efforts, there is still uncertainty among some animal care committee members about how to apply such guidance in the context of wildlife research. Typically, members are less acquainted with evaluating field methods, especially under the constraints of an approval system primarily focused on laboratory research animals.

Institutional animal care approvals can sometimes be sidestepped inappropriately. For example, Hervieux and colleagues [23, 24] conducted what they referred to as “experimental” killing of 733 wolves (*Canis lupus*) via aerial gunning and strychnine poisoning to measure the response of threatened caribou (*Rangifer tarandus*) prey populations in Alberta, Canada. Despite the involvement of several academic authors, there were no statements that an animal care approval was provided by their academic institutions, which would have very likely rejected such inhumane methods [25]. In another example, 1,966 lethal neck snares for coyotes (*Canis latrans*) were deployed to test effects of predator removal on caribou calf survival in Newfoundland, Canada [26]. Again, the authors provided no reference to an animal care approval. Notably, killing neck snares have been deemed inadequate to render canids unconscious consistently and humanely [27]. Although the use of neck snares is legal in Canada, such legality should not trump animal welfare principles.

## Towards editorial reform

Recognizing the shortcomings of other layers of oversight, we identify a pathway that could harness the influence of editors to prevent harm to wildlife and associated research processes. Because publishing comprises the central reward system of scientists, journals occupy a powerful position to standardize and improve conduct of researchers [4, 5]. To assess the potential for change, we scored 206 journals that commonly publish wildlife research to survey and score the presence and comprehensiveness of animal care policies (which govern the care and use of research animals) in journals. We expose troubling patterns but identify clear opportunities for reform. Additionally, we offer a new and practical resource: a policy template for animal care that meets minimal expectations for journals to consider when updating their animal care guidelines.

To examine animal care policies, we searched Institute for Scientific Information (ISI) Web of Science Journal Citation Reports year 2016 for all “Biodiversity Conservation” and “Zoology” journals to collate a broad sample of wild-animal–related journals. Focusing on vertebrates (the subphylum most commonly the focus of animal welfare), we excluded journals specializing in invertebrates. We added one journal not yet indexed (*Canadian Wildlife Biology and Management*), plus 14 interdisciplinary journals (e.g., *Science*, *Nature*) that commonly publish wildlife studies (see full list of journals uploaded with data). We searched “Instructions for Authors” or similarly titled documents or sections of websites across journals (*n* = 206). One person (KF) scored each on seven criteria guided by similar reviews of biomedical journals [4, 5]. Two criteria were coded as present or absent, and five were graded on strength of compliance language using the National Institute of Health’s interpretation of “must,” “should,” and “may” ([22]; S1 Text). Criteria were assigned 0 if absent from journal policies, 1 if their policies were suggestions, 2 if recommendations, and 3 if requirements. When journal instructions explicitly stated that authors must, should, or may follow external guidelines (*n* = 59 of 206) or publishing house guidelines (*n* = 29 of 206), we scored those external and publishing house guidelines for all criteria but one (criterion 7, which was journal-specific; S1 Text). We then incorporated these external and publishing house guideline scores into the focal journal scores using a compliance-language hierarchical approach (S1 Text). Data collection occurred during the period of October to December, 2017.

**Fig 1:**
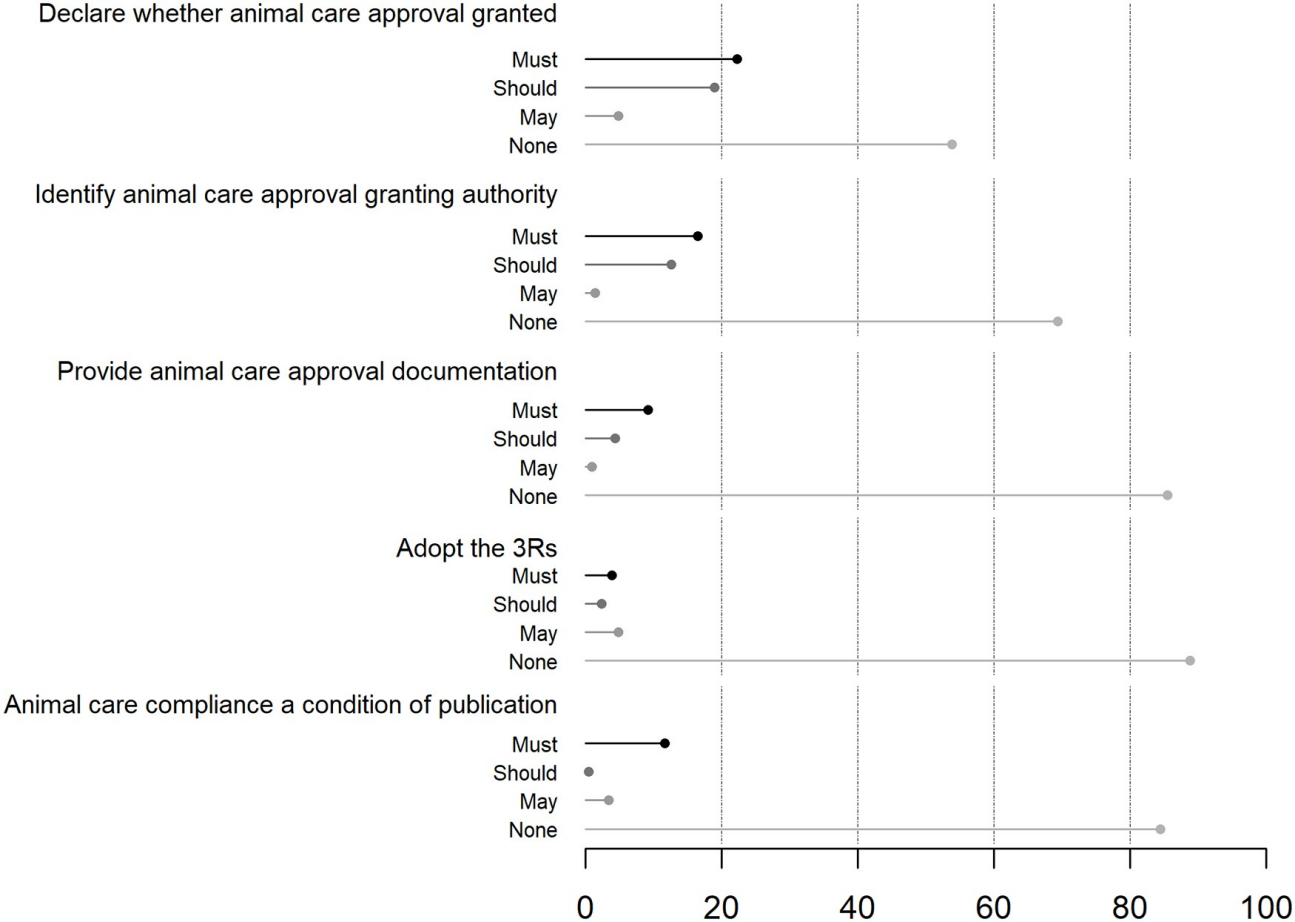
Presence and strength of compliance language in animal care policies across 206 journals that commonly publish wildlife research. 3R, replacement, reduction, refinement.

Journals varied considerably in their inclusion of criteria but collectively lacked fundamental safeguards against the mistreatment of wildlife. Of the 206 journals in our data set, one-third (*n* = 70) had no animal care policy. Of journals that had animal care policies, just 22% (*n* = 30, or 15% of total) had a statement related to best practices for animal care during fieldwork. The strength of the policy also varied; for each compliance-language criterion, journals that used “must” always constituted less than 34% of journals with animal care policies (*n* < 46, or <23% of total). The strongest language was most consistently found for the criterion requiring authors to state that an animal care approval was granted, yet only 34% of journals with animal care policies stated it “must” be followed (*n* = 46, or 22% of total). In addition, only 14% of journals with animal care policies (*n* = 19, or 9% of total) required authors to provide documentation of such an approval. Only 6% of journals with animal care policies (*n* = 8, or 4% of total) required that authors “must” adopt the 3R tenet of replacement (avoiding or replacing the use of animals), reduction (in number of animals used per study), and refinement (of methods to reduce suffering and improve welfare; Fig 1)—a central paradigm in the treatment of study animals [28].

**Fig 2:**
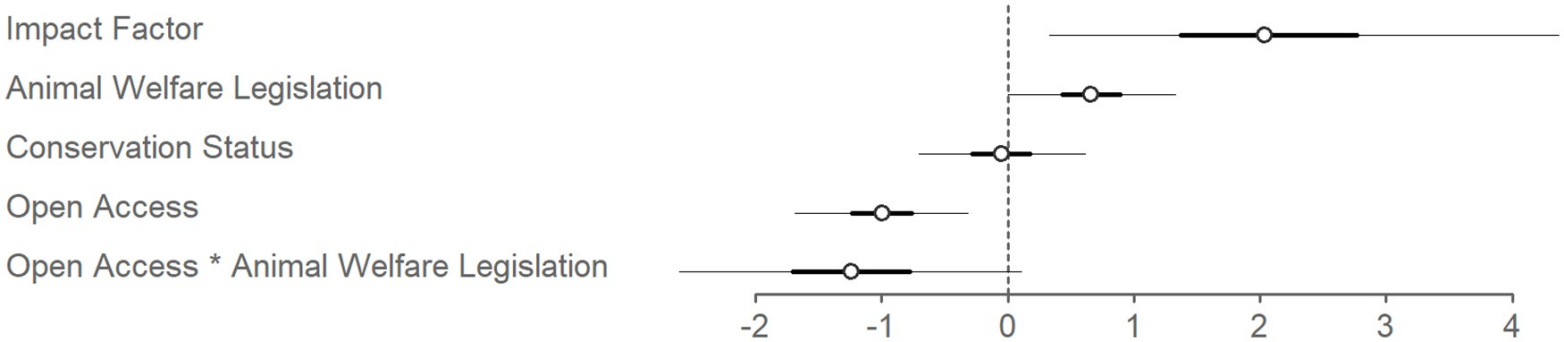
Association between journal characteristics and presence of animal care policies across 206 journals that commonly publish wildlife research. Coefficients shown are odds ratios from a logistic model, with thick and thin bars representing 50 and 95% confidence intervals, respectively. X scale unit is per 2 SDs of predictor.

We found associations between journal characteristics and the presence of animal care policies. Journals based in countries that endorse animal welfare law (Animal Protection Index; S1 Text) were more likely to have animal care policies (Fig 2). We encourage journals from countries without such legislation to adopt a best global practice to address this gap. We likewise found that journals with higher impact factors were more likely to have animal care policies (Fig 2). Although the association is not likely causative, lower-ranking journals without animal care policies may nonetheless be motivated to emulate what is perceived as best practice. In a complementary way, higher-impact journals with animal care policies can use their influence to advocate for change.

Contrary to our predictions and previous findings related to biomedical journal policies [4, 29], we found a negative association between open-access (OA) status and presence of animal care policy (Fig 2). We had reasoned that widely and readily accessible content would promote cautious editorial policy that avoided criticism from a potentially broad readership. Although we are uncertain why this differing pattern exists, we call for these OA journals without policies to adopt them rapidly.

The concerns of conservationists and animal welfarists sometimes fail to align. Across our data set, conservation orientation among journals had no effect on animal care policies (Fig 2) (see S1 Text for “conservation oriented” definition). A common contention is that animal welfare and conservation are incompatible because what is beneficial for conservation is not always so for individual animals and vice versa [10]. Conservationists often prioritize the integrity of populations, whereas animal welfare scientists primarily express concern about the suffering individual animals can endure [8, 10]. It has been suggested that “biologists recognize that conservation is engaged in the protection of the integrity and continuity of natural processes, not the welfare of individuals” [7]. However, employing invasive and lethal research methods in the name of conservation has raised important considerations about the welfare of individuals [12, 25]. Furthermore, failing to address the welfare of animals may ultimately jeopardize the integrity of wildlife populations and the continuity of natural processes [10]. Although the dichotomies between these two fields exemplify fundamental difficulties in harmonizing them, conceptual links exist [30]. Emerging from these linkages, “Compassionate Conservation” is a movement that diminishes approaches that intentionally and unnecessarily harm wildlife individuals while aligning with critical conservation goals [11]. Given that the health of populations and their constituent individuals are linked, conservation journals could contribute to momentum towards uniting conservation and animal welfare with robust animal care policies.

We found that author instructions were inconsistently articulated to readers. Animal care policies were often (36%) a hybrid format of external, publishing house, and journal guidelines. Confusion as to which guidelines require compliance might be compounded by a patchwork of different compliance language designed to enforce them. Indeed, our assignment of compliance-language scores was not straightforward because synonyms were used across journals, subjecting instructions to variable interpretation. Notably, gaps in compliance and apparent author confusion have been reported for other dimensions of journal policies, not just animal care [2]. Careful composition of journal policies explicitly posed as requirements could close loopholes that potentially accommodate sloppy reporting [31]. Simply put, policies posed as suggestions or recommendations lack force. Accounting for this reality and the criteria we surveyed, we propose a minimum template for mandatory animal care policy prescriptions that can be adapted to a wide variety of journals that contain wildlife research (Box 1). These guidelines, which we label Animals in Research: Reporting On Wildlife (ARROW, following ARRIVE guideline nomenclature; [32]), are not only realistic for journals to adopt, editors to enforce, and authors to comply with but also comprise a coherent baseline for journals currently lacking animal care policies (*n* = 70, or 34% of journals examined) upon which to build.

**Box 1. Recommended minimum requirements for animal care policies in journals that publish research on wildlife (“ARROW guidelines”)**

In the text of the manuscript, supplementary information, and/or cover letter, authors must

- state that they have obtained an institutional animal care approval and cite documentation of such an approval (including relevant application number to support tracking)
- state that they have complied with the relevant national, international, and institutional guidelines regarding animal care, naming them
- state that they have complied with, and cite, animal care legislation in the countr(y/ies) where their research was conducted
- state that they took all measures possible to follow the 3R tenets and describe such measures
- for research involving wildlife (captive or in natural settings), state that they have followed taxon-specific guidelines for the ethical treatment of the taxa of study and cite such guidelines

Failure to comply with journal policies on the care and use of animals will result in manuscript rejection. Editors maintain the discretion to reject work that imposes harm to research animals.

## Other considerations towards reform

Although alleviating weaknesses and disparities within and among laws, regulations, codes, and guidelines at various layers of oversight might appear to be an onerous pursuit, we offer some considerations towards improvement. First, we recommend that animal care committees have both a wildlife veterinarian and wildlife biologist formally available for consultation [17]. The knowledge of these field-experienced professionals must be relevant to the species and subject addressed by the research. By harnessing their knowledge of the physiology of pain and suffering, wildlife veterinarians can aid in assessing the humaneness of research methods in the field. Even where guidelines are endorsed by animal care committees (e.g., American Veterinarian Medical Association Guidelines on Euthanasia), veterinarians can aid in enforcing them. Wildlife biologists can likewise lend their expertise on techniques often used to study free-ranging species, addressing uncertainty among other animal care committee members to evaluate methods with which they might not be acquainted. Notably, the *Guide for the Care and Use of Laboratory Animals* [22] encourages animal care committees engaged in the review of field studies to consult with a qualified wildlife biologist. This ought to be a requirement. Submission of wildlife-specific animal use applications could also be required of authors by institutions. As an example, the University of Montana requires that a “Wildlife Animal Use Protocol” be submitted for any study conducted on free-living wild animals in their natural habitat [33]. Another contribution wildlife biologists can make involves external permits. Whereas animal care committees are primarily charged with assessing the welfare of individual research animals, a variety of international, national, state/provincial/territorial, and local permits exist to inform animal care committees that population-level impacts are acceptable and relevant laws are followed [34]. Wildlife biologists can render their expertise to assess whether researchers have obtained all necessary wildlife research permits. This interaction aligns with the emerging understanding that healthy populations and the welfare of animals that comprise them are linked [10]. With these additional contributions from veterinarians and wildlife biologists, animal care committee approvals might provide a higher level of early (upstream) safeguard against unnecessary suffering of wildlife used in study.

When legal and institutional levels of oversight fail wildlife, editorial policy can provide an additional and potentially robust bulwark against harm. Our results and case studies, however, suggest animal care policies are not only inconsistent across journals but also insufficiently enacted to ensure welfare across taxa. Vast differences between biomedical and wildlife research animals [6] must be considered in journal animal care policies. Owing to an environment that can never be fully controlled (or even observed, in the case of wildlife implanted with telemetry or other remote measuring devices), journal policies tailored to biomedical research animals will not guarantee the welfare of animals studied in the wild. Although taxon-specific organizations have created field-based animal care guidelines (e.g., [35]), they are not widely endorsed in journal policies. While we agree taxon-specific guidelines ought to be endorsed, we suggest that they be approached critically. For example, some guidelines (e.g., [35]) have proposed submersion trapping (death by drowning-induced hypoxia) to kill mammals, which does not align with criteria indicating relative humaneness of trap performance [27].

Even the best animal care policies can fail wildlife if not enforced [36]. Editorial implementation of the Animals in Research: Reporting In Vivo Experiments (ARRIVE) reporting guidelines, for example, has not been effective because authors, reviewers, and journal editors have frequently ignored them [37]. Accordingly, apparent violations of journal policy and subsequent failure of editorial oversight requires whistleblowing by concerned members of the public, scientists (e.g., [25]), and editors (e.g., [38]) to prevent abuse. Costello et al. [38] set the agenda for impeccable editorial discretion when they rejected wildlife studies they deemed inhumane, even though the studies had been institutionally approved. For manuscripts with potentially problematic treatment of animals, editors could solicit specialized reviewers to examine animal care statements of authors, perhaps also requesting from authors the associated animal care committee proposals and approvals for evidence of compliance. At minimum, editors could require authors to submit detailed documentation of approval from an animal care committee (Box 1)—such a requirement was absent from 78% of journals with animal care policies (*n* = 106; or 85% of total, *n* = 176). Reviewers should also be involved in assessments of not only science but also animal care. In the most basic sense, whether or not authors adequately provided animal care information can constitute one of the standard questions reviewers must answer during reviews.

Researchers and members of scholarly societies, many of the latter publishing their own journals, can also lobby for change. If members are concerned and aware of the potential for journals to influence the behavior of wildlife researchers, and thus the animals they study, they can call on societies to ensure their policies, as well as the policies of their journals, are adopting best practices. Researchers can play a role via self-regulatory compliance. Individual behavior is shaped by values, laws, editorial policies, and membership in scholarly societies as well as the community norms that emerge from the interactions of these influences. Although editorial oversight occurs downstream from study design and interactions with animals, publication reform that standardizes animal care expectations will prompt researchers to conform to best welfare practices with knowledge that such is a condition of publication (notably, a criterion that we suggest journals adopt; Box 1). This aligns with an element of a “Nine R Theory” (see [39]), which offers “Refusal” as a means to gauge whether the harm is worth the gain during planning stages of research. Animal Refusal “rejects the initial animal research plan completely to prevent animals from suffering futile harm or harm not worth the gain,” sending researchers back to the drawing board. Although transformation will take time, we envision a shift in researcher culture towards greater attention to animal care, similar to recent changes towards open data, reproducibility, and co-authorship expectations. Even if journals do not yet demand detailed evidence of best-practice animal care, scientists can practice and report on criteria we outline in ARROW guidelines.

The development and maintenance of nonlegislated animal welfare standards rely heavily on voluntary compliance with codes of practice (e.g., [40]). Codes often specify rules of protocol as well as the moral responsibilities of professionals. Unfortunately, codes are of limited value if not enforced or can oversimplify moral requirements, causing professionals to suppose mistakenly that they have satisfied moral requirements simply by following the rules of the code [41]. Surveillance of, and reporting on, suspected animal care violations (e.g., [26]) would further ensure the maintenance of professional, self-regulatory compliance as well as wildlife welfare.

Ethical conduct in science occupies a significant place in the public’s perception of the role of scientists [42], affording social license to the research process. At the same time, public attitudes towards animals are often a driving force behind improvements in animal-related policy [43]. For example, societal objection to the use of hot-iron identification branding of pinnipeds encouraged the Australian government to ban the research method after video footage of branding was shown in the media. Despite all ethical and legislative permissions being granted and all researchers working within the prescribed permissions’ boundaries, the research was halted [14]. Invasive research has indeed marshalled compelling public opposition (e.g., [44, 45]) questioning the moral legitimacy of research that knowingly harms research animals. Use of wildlife to generate new knowledge hinges on implicit consent from the majority of society. Because the public generally cherishes wildlife, mistreating them jeopardizes the privilege of trust in the scientific endeavor.

## Straightforward corrective action by journals

Scientific publishing is rapidly adapting to, and shaping, interests among research communities, governments, funders, and the public. Emphasis on upholding ethical standards in science and its publication process receives high-profile attention and debate, exposing conduct ripe for reform. Responding to this opportunity, we have provided a practical resource (Box 1—ARROW guidelines) and ideally ignited a discussion towards ethically attuned animal care policies and practice to protect the welfare of wildlife. Data deposited in the Dryad repository: https://doi.org/10.5061/dryad.c53k1d6 [46].

## Supporting information

S1 Text Data analyses with associated tables and figures. (DOCX)

## Abbreviations

3R: replacement, reduction, refinement
ARRIVE: Animals in Research: Reporting In Vivo Experiments
ARROW: Animals in Research: Reporting On Wildlife
CCAC: Canadian Council for Animal Care
ISI: Institute for Scientific Information
OA: open access
