# Correction: Publication reform to safeguard wildlife from researcher harm

**DOI:** 10.1371/journal.pbio.3000752

**Published:** 2020-05-18

**Authors:** Kate A. Field, Paul C. Paquet, Kyle Artelle, Gilbert Proulx, Ryan K. Brook, Chris T. Darimont

In this work [1], the following sentence within the Introduction was stated incorrectly:

Although recent revisions to the Guide incorporated consultation with wildlife biologists, a recent assessment considered the consultation to have occurred in a “cursory and broad manner” [6].

The original intent of the statement was to indicate that wildlife biology was not addressed in The Guide to the Care and Use of Laboratory Animals until recently, and that reference to wildlife biology occurred in a cursory and broad manner. The statement should be revised as follows:

Although recent revisions to the Guide addressed wildlife biology, a recent assessment considered this to have occurred in a “cursory and broad manner” with no evidence of consultation with wildlife biologists [6].
