# Supplementary material for: Publication reform to safeguard wildlife from researcher harm
Source: PLoS Biol. 2019 Apr 11;17(4):e3000193. doi: 10.1371/journal.pbio.3000193 (PMC6459470; doi:10.1371/journal.pbio.3000193)
Supplement: S1 Text — (DOCX) [file pbio.3000193.s001.docx]

**Supporting Information (S1 Text)**

We evaluated intra- and inter-observer precision in scoring. An external participant provided the lead scorer (KF) with a random 10% subset of journals to re-score, and another participant a non-overlapping 10% subset to score. The same external participant scored both these exercises for agreement. We excluded criteria with agreements lower than 75% (S5 Table in S1 Text). Across criteria with agreements higher than 75%, intra-observer rescores matched original assessments 94% of the time. Interobserver scores matched original assessments 86% of the time (S4 Table in S1 Text).

We conducted a logistic regression analysis to test for associations between the presence of any animal care policy and journal characteristics (impact factor, open-access status, animal welfare legislation in country of the journals’ headquarters, and whether the journal was conservation-oriented). We centered all predictors (subtracted the mean from each observation) and scaled (divided by 2 SDs) [1]. We also used a Poisson regression analysis to test for associations with number of criteria detected among journals that had an animal care policy and the same journal characteristics. For the latter, criteria were considered fulfilled if a journal received any score other than “none”. We considered journals conservation-oriented if the content and language of the ‘aim and scope’ reflected a conservation focus or if ‘conservation’ appeared in the journals’ title and/or ‘aims and scope’ (e.g., “*Amphibia-Reptilia* is a leading European multi-disciplinary journal devoted to most of the aspects of herpetology: ecology, behaviour, evolution, conservation, physiology, morphology, paleontology, genetics, and systematics”). We considered journals to be open access only if exclusively so (e.g., no ‘hybrid’ models). We categorized countries as “full” or “partial” application of legislation against causing animal suffering according to the Animal Protection Index by World Animal Protection (S6 Table in S1 Text; that is, whether animal protection laws that prohibit causing animal suffering either by a deliberate act of cruelty or by a failure to act are fully or partially endorsed; <https://api.worldanimalprotection.org/>). There were no countries in our dataset that Animal Protection Index classified as fully lacking animal protection legislation. We excluded journals from this analysis with headquarters located in countries not indexed by the Animal Protection Index (n = 8, S6 Table in S1 Text).

For the Poisson model, we excluded two criteria that were co-dependent (*Does the journal ask that authors specify the institutional authority that granted animal care licenses or permits?*; and *Does the journal ask that authors provide animal care license or permit numbers?;* were contingent upon *Does the journal ask that authors state whether an animal care permit or approval was granted?*).

We tested for correlation between variables using Pearson correlation coefficient. No two variables were correlated (S1 Table). We computed the variance inflation factors (VIF) for each model to test for multicollinearity. We did not detect multicollinearity (S2 Table). The mean and median impact factor of journals was 2.03 and 1.2, respectively, with journals removed that had not yet had an impact factor assigned. All analyses were conducted in R Version 3.4.2 [2].

**S1 Table.** Pearson’s correlation coefficient for each pair of independent variables.

| Variables | | *r* | *P* |
| --- | --- | --- | --- |
|  | Open access * welfare legislation | -0.04 | 0.62 |
|  | Open access * conservation oriented | 0.01 | 0.90 |
|  | Open access * impact factor | -0.06 | 0.37 |
|  | Welfare legislation * conservation oriented | -0.11 | 0.11 |
|  | Impact factor * welfare legislation | 0.03 | 0.67 |
|  | Impact factor * conservation oriented | -0.08 | 0.25 |

**S2 Table.** Variance inflation factor values for logistic and Poisson regression analyses.

| Model | | Variables | VIF |
| --- | --- | --- | --- |
|  | Logistic regression | Impact factor | 1.014 |
|  |  | Open access | 1.028 |
|  |  | Welfare legislation | 1.054 |
|  |  | Conservation oriented | 1.023 |
|  |  | Welfare legislation * open access | 1.026 |
|  | Poisson regression | Impact factor | 1.013 |
|  | | Open access | 2.636 |
|  |  | Welfare legislation | 1.384 |
|  |  | Conservation oriented | 1.065 |
|  |  | Welfare legislation * open access | 2.819 |

Regardless of compliance language and across criteria that were not co-dependent, most (72%; n = 98) journals that had animal care policies fulfilled less than two (of 4; 50%) criteria (Fig A. in S1 Text). Taxon- (e.g., Acta Chiropterologica) or subphylum vertebrata class-specific (e.g., Journal of Mammalogy) comprised 20% (n = 42) of journals. Books that were referenced in journal guidelines (n = 2 journals) were not scored.


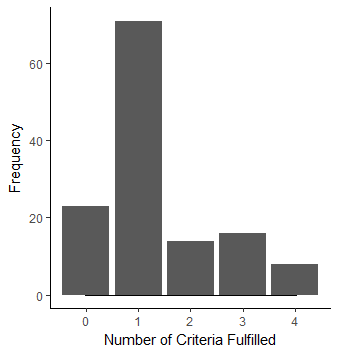


**S1 Fig.** Number of criteria fulfilled among journals. Regardless of compliance language and across criteria that were not co-dependent (criteria 2, 3, 4, 5; S3 Table in SI Text), most (72%; n = 98) journals that had animal care policies fulfilled less than two criteria.


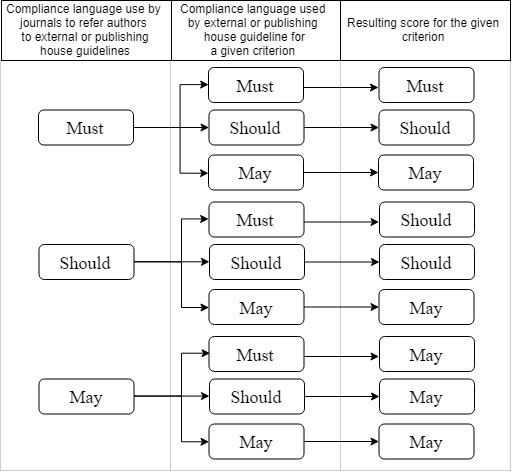


**S2 Fig.** Hierarchical approach to incorporating external and publishing house guideline scores into final journal scores based on compliance-language used 1) by a journal to direct authors to such guidelines, and 2) within such guidelines.

**S3 Table.** Scoring criteria for animal care policies in journals.

| Criteria | | Categorical Score |
| --- | --- | --- |
| 1 | statement regarding animal care policy | Yes, No |
| 2 | statement related to best practices for wildlife *in situ* |  |
| 3 | statement regarding the adoption of the 3Rs (Replacement, Reduction and Refinement) | None, May, Should, Must^^[[1]](#footnote-1)^^ |
| 4 | requirement that authors state that an animal care permit or approval was granted |  |
| 5 | requirement that authors identify the institutional authority that granted animal care permit or approval |  |
| 6 | requirement that authors provide animal care permit or approval number |  |
| 7 | statement informing authors that compliance with animal care journal policies is a condition of publication |  |

**S4 Table.** Inter- and intra-observer agreement across criteria.

| Criteria | | Intra-observer agreement (%) | Inter-observer agreement (%) |
| --- | --- | --- | --- |
| 1 | statement regarding animal care policy | 100 | 95 |
| 2 | statement related to best practices for wildlife *in situ* | 95 | 90 |
| 3 | statement regarding the adoption of the 3Rs (Replacement, Reduction and Refinement) | 95 | 85 |
| 4 | requirement that authors state whether an animal care permit or approval was granted | 85 | 75 |
| 5 | requirement that authors identify the institutional authority that granted animal care licenses or permits | 95 | 95 |
| 6 | requirement that authors provide animal care license or permit numbers | 90 | 90 |
| 7 | statement informing authors that compliance with animal care journal policies is a condition of publication | 95 | 75 |
|  | **Average agreement** | 94 | 86 |

**S5 Table.** Inter- and intra-observer agreement across criteria that were excluded due to low inter-observer agreement.

| Criteria | | Intra-observer agreement (%) | Inter-observer agreement (%) |
| --- | --- | --- | --- |
| 1 | statement requiring that authors comply with relevant animal care legislation | 95 | 60 |
| 2 | statement requiring that authors comply with relevant national, international or institutional guidelines regarding animal care | 95 | 55 |
| 3 | statement requiring that authors state that they complied with relevant animal care legislation | 100 | 60 |
| 4 | statement requiring that authors state that they complied with national, international or institutional guidelines on animal care | 95 | 50 |
|  | **Average agreement** | 96 | 56 |

**S6 Table.** Animal Protection Index rankings of countries of journal headquarters. Data from <https://api.worldanimalprotection.org>

| Country | Animal Protection Index Ranking for “Laws against causing animal suffering” |
| --- | --- |
| Australia | Legislation |
| Austria | Legislation |
| Belgium | Not ranked |
| Brazil | Legislation with partial application |
| Bulgaria | Not ranked |
| Canada | Legislation |
| Chile | Legislation |
| China | Legislation with partial application |
| Colombia | Legislation |
| Czech Republic | Not ranked |
| Denmark | Legislation |
| England | Legislation |
| Finland | Not ranked |
| France | Legislation with partial application |
| Germany | Legislation |
| Hungary | Not ranked |
| Italy | Legislation |
| Japan | Legislation |
| Kenya | Legislation |
| Mexico | Legislation with partial application |
| Netherlands | Legislation |
| New Zealand | Legislation |
| Pakistan | Legislation with partial application |
| Poland | Legislation |
| Romania | Legislation |
| Russia | Legislation with partial application |
| Singapore | Not ranked |
| Slovakia | Not ranked |
| South Africa | Legislation with partial application |
| Spain | Legislation with partial application |
| Switzerland | Legislation |
| Taiwan | Not ranked |
| Turkey | Legislation |
| USA | Legislation with partial application |

**References**

1. Gelman A. Scaling regression inputs by dividing by two standard deviations. Stat Med. 2008;27: 2865-2873.
2. R Core Team. R: A language and environment for statistical computing. Foundation for Statistical Computing, Vienna, Austria. 2017. Available from: <https://www.R-project.org/>.
3. National Research Council [Internet]. Guide for the care and use of laboratory animals. c2011. [cited 2018 Dec 19]. Available from: <https://grants.nih.gov/grants/olaw/guide-for-the-care-and-use-of-laboratory-animals.pdf>

1. “Must indicates actions that…[are] imperative and mandatory duties or requirements for providing humane animal care and use. Should indicates a strong recommendation for achieving a goal; however…individual circumstances might justify an alternative strategy. May indicates a suggestion to be considered.” [3] [↑](#footnote-ref-1)
